# Genome-wide association study reveals that different pathways contribute to grain quality variation in sorghum (*Sorghum bicolor*)

**DOI:** 10.1186/s12864-020-6538-8

**Published:** 2020-01-31

**Authors:** Wilson Kimani, Li-Min Zhang, Xiao-Yuan Wu, Huai-Qing Hao, Hai-Chun Jing

**Affiliations:** 1 Key Laboratory of Plant Resources, Institute of Botany, Chinese Academy of Science, Beijing, 100093 China; 2 University of Chinese Academy of Sciences, Beijing, 100049 China; 3 Engineering Laboratory for Grass-based Livestock Husbandry, Institute of Botany, Chinese Academy of Sciences, Beijing, 100093 China

**Keywords:** Sorghum, Grain quality, Genome-wide association study, Amino acids, Starch, Tannins

## Abstract

**Background:**

In sorghum (*Sorghum bicolor*), one paramount breeding objective is to increase grain quality. The nutritional quality and end use value of sorghum grains are primarily influenced by the proportions of tannins, starch and proteins, but the genetic basis of these grain quality traits remains largely unknown. This study aimed to dissect the natural variation of sorghum grain quality traits and identify the underpinning genetic loci by genome-wide association study.

**Results:**

Levels of starch, tannins and 17 amino acids were quantified in 196 diverse sorghum inbred lines, and 44 traits based on known metabolic pathways and biochemical interactions amongst the 17 amino acids calculated. A Genome-wide association study (GWAS) with 3,512,517 SNPs from re-sequencing data identified 14, 15 and 711 significant SNPs which represented 14, 14, 492 genetic loci associated with levels of tannins, starch and amino acids in sorghum grains, respectively. Amongst these significant SNPs, two SNPs were associated with tannin content on chromosome 4 and colocalized with three previously identified loci for *Tannin1*, and orthologs of *Zm1* and *TT16* genes. One SNP associated with starch content colocalized with sucrose phosphate synthase gene. Furthermore, homologues of *opaque1* and *opaque2* genes associated with amino acid content were identified. Using the KEGG pathway database, six and three candidate genes of tannins and starch were mapped into 12 and 3 metabolism pathways, respectively. Thirty-four candidate genes were mapped into 16 biosynthetic and catabolic pathways of amino acids. We finally reconstructed the biosynthetic pathways for aspartate and branched-chain amino acids based on 15 candidate genes identified in this study.

**Conclusion:**

Promising candidate genes associated with grain quality traits have been identified in the present study. Some of them colocalized with previously identified genetic regions, but novel candidate genes involved in various metabolic pathways which influence grain quality traits have been dissected. Our study acts as an entry point for further validation studies to elucidate the complex mechanisms controlling grain quality traits such as tannins, starch and amino acids in sorghum.

## Background

With the increasing demand for healthy and nutritious food, developing crop varieties with enhanced grain quality is an important target for many breeding programs. Sorghum (*Sorghum bicolor*) is a major cereal crop which provides food for over half a billion people in the arid and semi-arid tropics of Africa and Asia, which manage to produce high yield under drought and high-temperature stress prevalent in these regions. Sorghum grain is a source of carbohydrates, minerals, proteins, vitamins, and antioxidants [1]. Understanding the natural variation and genetic architecture of grain quality traits in sorghum is a first step towards improvement of the nutritional quality through conventional and molecular breeding.

Grain quality is determined by the biochemical and physical characteristics of the grain. It varies among cereal crops and diverse germplasm, but in general, cereal grains mainly contain starch, protein and fat. Some sorghum germplasms contain unique phenolic compounds, including condensed tannins. Starch is the most important component which provides energy to humans and livestock and accounts for ∼70% of dry grain weight in cereals [2]. There are two types of starch in cereal grains, including amylose and amylopectin. And the ratio of these two starches plays an essential role in grain structure and quality. Starch biosynthesis and assembly in cereals are catalyzed by various vital enzymes, including ADP-glucose pyrophosphorylases (AGPase), soluble starch synthase (SS), starch branching enzyme (SBE), starch debranching enzyme (DBE) and granule-bound starch synthase (GBSS) [3]. Mutations which cause changes in activities of these enzymes and subsequent variation in starch quality and quantity have been discovered. For instance, in maize, *shrunken1* and *amylose extender1* affect the amylose content in starch granules [4]. The s*ugary* mutants in maize are used to produce sweet maize with increased sucrose content and reduced concentration of amylopectin [5]. In sorghum, mutants of *waxy* gene that encodes GBSS, have little or no amylose, thus increased protein and starch digestibility [6]. The s*ugary* mutants which contain high water-soluble carbohydrates in the endosperm have also been characterized in sorghum [7].

Grain quality traits such as digestibility and nutritional value depend heavily upon the content of the cereal proteins, which are primarily attributed to their amino acid composition. Cultivated sorghums have limited levels of threonine (Thr) and lysine (Lys) [8], which are two of the nine essential amino acids for humans and animals. Besides the primary role of protein synthesis, amino acids are precursors for osmolytes, hormones, major secondary metabolites and alternative energy source [9]. Also, amino acids are crucial for seed development and germination as well as plant stress response. To date, the amino acid metabolism pathways have been well studied, and key genes regulating these pathways have been identified in *Arabidopsis* [10, 11], tomato [12], soybeans [13], rice [14] and maize [15]. Among the well characterized genes are *Opaque-2* (*O2*), *floury-2* and *high-lysine*, whose mutants have high lysine concentrations [15]. These mutations could be used to enhance the nutritional value of cereal grains. Although the lines with high lysine have continued to be used in research, they are yet to be commercially used except for quality protein maize (QPM) [16]. The major setback of high lysine mutations in cereals is their adverse effects on agronomic performance especially low yield. Identification of alternative genes that would enhance the grain nutritional quality without compromising on the yield and in-depth understanding of amino acids metabolism are essential steps in the development of sorghum grains with high-quality proteins.

Flavonoids including flavonols, anthocyanins and proanthocyanidins (also called condensed tannins), are secondary metabolites in higher plants known for the pigmentation in flowers, fruits and seeds [17]. Flavonoids significantly contribute to human health due to their antioxidant capacity and radical scavenging functions [18]. In plants, condensed tannins protect against insects, birds, herbivores, cold tolerance, bacterial and fungal infections. Pharmacological studies have shown that tannins have considerable health-promoting properties. Therefore, they may be potentially used as nutraceuticals or dietary supplements [19].

The genetic control and biochemical pathways for condensed tannins have been extensively studied in maize and *Arabidopsis* [20]. Recently, *Tannin1*, a gene underlying the *B2* locus in sorghum and encoding a WD40 protein, was cloned [21]. It is a homologue to *TRANSPARENT TESTA GLABRA 1* (*TTG1*), a regulator of proanthocyanidins in *Arabidopsis*. Furthermore, an MYB transcription factor, *Yellow seed1* (*Y1*) which controls pericarp pigmentation and 3-deoxyanthocyanidins accumulation in sorghum pericarp, has been cloned [21]. However, there still exists a significant gap in knowledge of the available diversity of tannins and the underlying genetic mechanisms.

GWAS has been proven to be a powerful tool in determining the genetic basis of complex traits in plants, including grain quality traits [7, 22–24]. It can evaluate several alleles at a single locus from natural populations to provide a higher mapping resolution as opposed to the linkage mapping which can only assess limited loci from biparental populations to capture narrow levels of allelic diversity [25]. In sorghum, using genotyping-by-sequencing data, GWAS has been used to identify QTLs for several grain quality traits including polyphenols [26], proteins and fat [7], minerals [27], amylose, starch, crude protein, crude fat, and gross energy [28]. Here we present the use of high-density re-sequencing data to characterize the population structure of 196 diverse sorghum accessions and to identify the genetic loci and candidate genes underlying natural variations of tannins, starch and amino acids in sorghum.

## Results

### Genetic structure and linkage disequilibrium of the assembled association panel

**Fig. 1:**
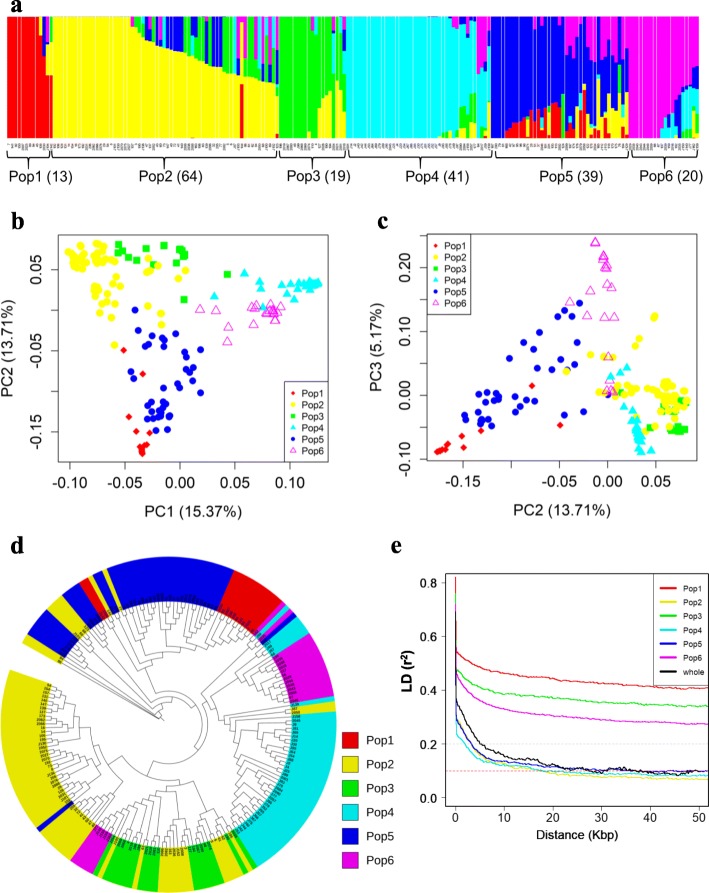
Population structure analysis of 196 diverse sorghum accessions using genome-wide SNPs. **a** Hierarchical organization of genetic relatedness of the 196 diverse sorghum lines. Each bar represents an individual accession. The six sub-populations were pre-determined as the optimum number based on ADMIXTURE analysis with cross-validation for K value from K = 2 to K = 10 using 841,038 unlinked SNPs (r^2^ < 0.8), distributed across the genome. Different colours represent different sub-populations. **b** A plot of the first two principal components (PCs) coloured by sub-populations. **c** PC2 vs PC3 coloured by sub-populations. **d** Phylogenetic tree constructed using the maximum likelihood method in SNPhylo. The colours are based on the six sub-populations from ADMIXTURE results. **e** Comparison of genome-wide average linkage disequilibrium (LD) decay estimated from the whole population and six sub-populations. The horizontal broken grey and red lines show the LD threshold at r^2^ = 0.2 and r^2^ = 0.1, respectively

Population structure was calculated with a filtered set of 841,038 SNPs. Six ancestral populations (later referred to as Pop1 to Pop6) were identified based on the K value corresponding to the lowest cross-validation error in the ADMIXTURE software [29] (Fig. 1a). Pop1 (*n* = 13) consisted mostly of improved lines of African origin. Pop2 (*n* = 64) and Pop3 (*n* = 19) showed a close relationship and consisted mostly of improved lines from at least 25 countries/regions. At least 80% of accessions in Pop4 (*n* = 41) were landraces from China. Pop5 was comprised of 69 and 31% improved lines and landraces, respectively, from USA (*n* = 11), Sudan (*n* = 8) and Ethiopia (*n* = 6). Pop 6 was composed of 14 landraces and 6 improved lines, with majority of Asian origin (Additional file 3: Table S1). We also performed Principal Component Analysis (PCA) to investigate the relationship amongst accessions in the diversity panel (Fig. 1b, c). PC1 to PC3 captured ~ 34.25% of the genetic variation. When the six sub-groups from ADMIXTURE were integrated into the PCA biplots of PC1 vs PC2 and PC2 vs PC3, three clusters consisting of two sub-populations each were observed, i.e. Pop2 and Pop3, Pop1 and Pop5, and Pop4 and Pop6 (Fig. 1b, c).

We further inferred the relationships amongst the six sub-populations by constructing a maximum likelihood tree using unlinked SNP markers by running DNAML programs in the PHYLIP integrated in SNPhylo [30] (Fig. 1d). The six sub-groups were in three major clades. Majority of accessions in Pop2 and Pop3 shared a clade, Pop4 and Pop6 shared another clade while Pop1 and Pop5 clustered into one clade. This suggests high genetic relatedness amongst genotypes within similar clades, resembling their differentiation in structure analysis and PCA (Fig. 1a, b and c).

Another way of exploring the genome landscape of a population for association mapping is the extent of LD decay as a function of the physical distance for all chromosomes. We estimated the extent of LD decay within the six sub-groups and the whole diversity panel using genome-wide SNPs. The LD decay rate significantly varied amongst the six sub-groups, and the LDs of Pop2, Pop4 and Pop5 decayed much faster than those of Pop1, Pop3 and Pop6 (Fig. 21d). The whole population showed a rapid decline in average LD with the increase in distance, where it decreased to r^2^ = 0.2 at ~ 8 kb distance, and reached to the optimum threshold value (r^2^ = 0.1) at ~ 40 kb (Fig. 21d).

**Fig. 2:**
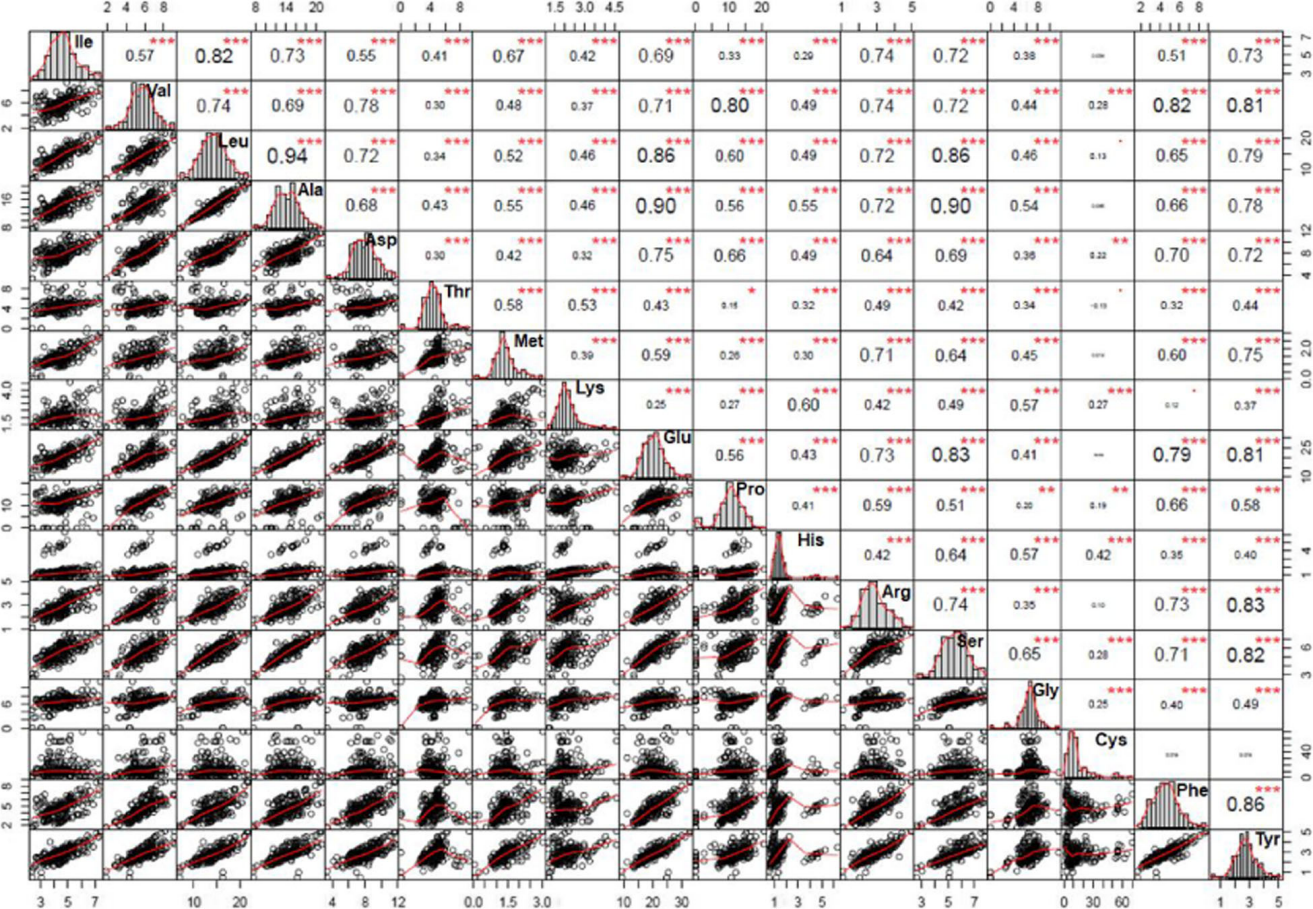
Variations and spearman’s correlations among 17 amino acids. The lower panel left of the diagonal is the scatter plots containing measured values of 196 accessions. The red line through the scatter plot represents the line of the best fit. Spearman’s correlation coefficients between amino acids are shown on the upper panel on the right of the diagonal. The correlation significance levels are *p = 0.05, **p = 0.01 and ***p = 0.001, and the size of the coefficient values are proportional to the strength of the correlation

### Natural variation of grain quality traits

To assess the extent of natural variation in grain quality traits in sorghum, we quantified tannin, starch and 17 amino acids levels from the flour of dry, mature sorghum grains from 196 diverse sorghum accessions (Additional file 4: Table S2). Tannin and starch levels were expressed as the percentage of dry grain weight and ranged from 1.2 to 2.2%, and 38.6 to 75.8%, respectively. Amino acid levels were expressed as nmol mg^− 1^ of dry grains flour. Among the 17 amino acids detected, Glu and Cys were the most abundant amino acids, and His and Met were the least abundant, with average relative compositions (absolute level/Total*100) of 16.15, 11.82, and 1.15%, 1.15%, respectively (Table 1). The relationships amongst amino acids were calculated using Spearman’s rank correlation method, and the results were visualized using PerformanceAnalytics package (Fig. 2). Amino acids dominantly showed positive correlations except only one weak negative relationship between Cys and Thr. Amino acids which are biologically related exhibited strong positive correlations. For instance, branched-chain amino acids (BCAA), Ile, Val and Leu, were highly correlated with r_sp_ values ranging from 0.6 to 0.82 for Ile vs Val and Ile vs Leu, respectively. Additionally, to uncover the regulators of amino acids in sorghum grains, we derived 44 more traits from absolute amino acids levels (detailed in methods; Additional file 5: Table S3) based on biological relationships amongst 17 amino acids and used them as phenotypes for GWAS.

**Table 1:** Summary statistics of tannins, starch and 17 amino acid contents measured in the association panel

Trait	Units	Absolute value (pmol ul^− 1^ mg^− 1^)				Relative value (% of total)
		Mean	SD	Minimum	Maximum	
Ala	nmol mg^−1^	14.38	2.56	7.60	21.07	11.45
Arg	nmol mg^−1^	2.84	0.69	1.09	4.96	2.26
Asp	nmol mg^−1^	7.95	1.54	3.36	11.69	6.33
Cys	nmol mg^−1^	14.83	13.56	0.05	70.56	11.82
Glu	nmol mg^−1^	20.27	3.95	9.44	32.92	16.15
Gly	nmol mg^−1^	6.49	1.44	0.05	11.49	5.17
His	nmol mg^−1^	1.45	0.93	0.60	6.32	1.15
Ile	nmol mg^−1^	4.48	1.02	2.40	7.42	3.57
Leu	nmol mg^−1^	14.41	2.79	7.02	21.79	11.48
Lys	nmol mg^−1^	2.09	0.59	1.16	4.60	1.67
Met	nmol mg^−1^	1.45	0.48	0.05	3.03	1.15
Phe	nmol mg^−1^	4.56	1.33	1.69	8.75	3.63
Pro	nmol mg^−1^	11.06	3.97	0.05	20.60	8.81
Ser	nmol mg^−1^	5.56	0.98	2.65	7.79	4.43
Thr	nmol mg^−1^	4.49	1.30	0.05	9.23	3.57
Tyr	nmol mg^−1^	2.81	0.72	0.42	5.08	2.24
Val	nmol mg^−1^	5.72	1.28	1.87	9.16	4.56
Starch	% dry grain weight	59.28	6.02	38.65	75.80	–
Tannin	% dry grain weight	1.48	0.24	1.16	2.24	–

**Fig. 3:**
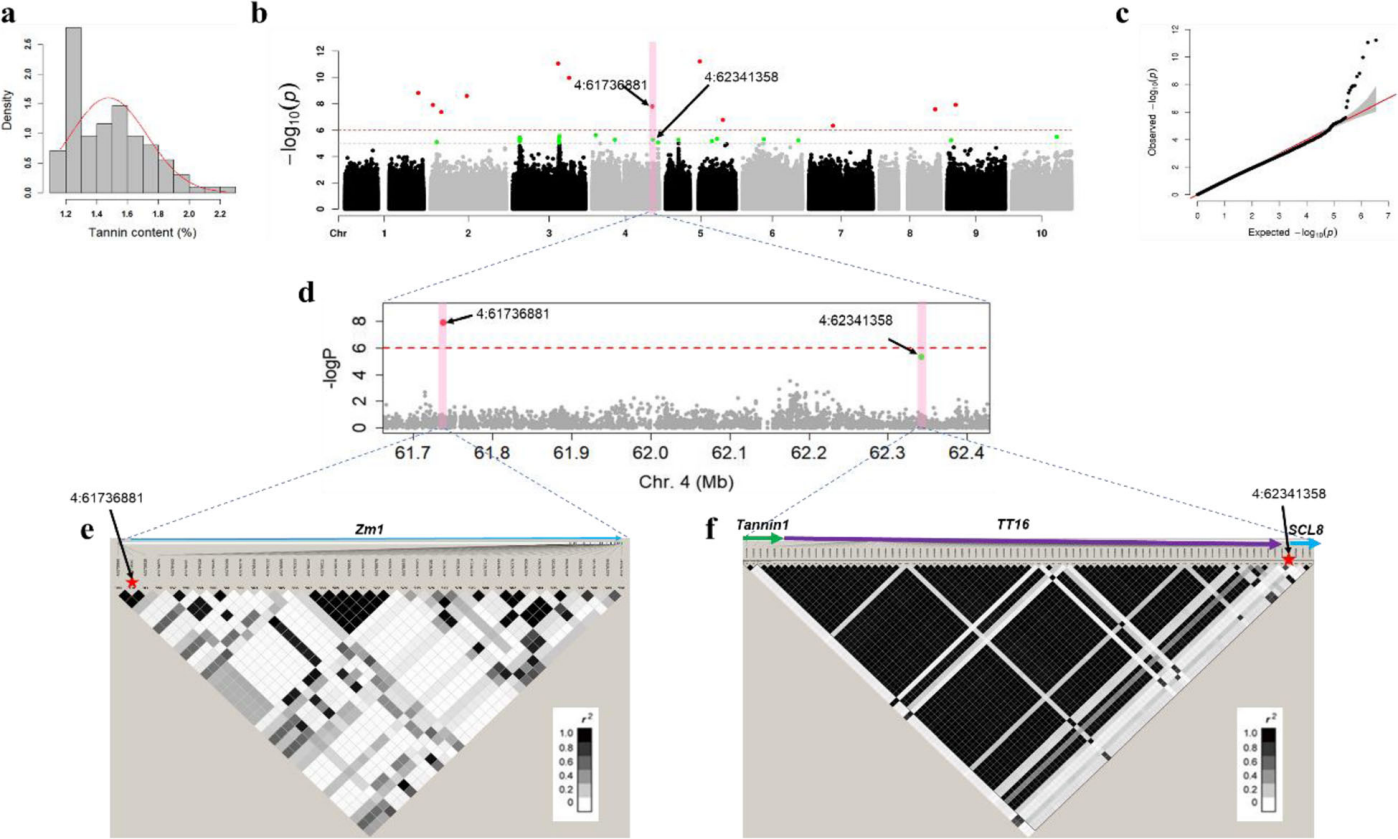
GWAS for Tannin levels in sorghum seed and direct hits to a priori candidate gene region. **a** Distribution of tannin content in 196 diverse accessions. **b** Manhattan plot for tannin content GWAS. Black arrows show associated SNPs located close to candidate genes. **c** Quantile-quantile plot for tannin content GWAS. **d** A close up of the significant association on chromosome 4. The broken red line represents the significance threshold. **e** and **f** LD blocks showing pairwise r^2^ values among all polymorphic sites in candidate genes region, where the intensity of the colour corresponds to the r^2^ value as indicated on the legend. Candidate genes *Zm1* (~ 61.7 Mb region), *Tannin1*, *TT16* and *SCL8* (~ 62.3 Mb region) are shown

**Fig. 4:**
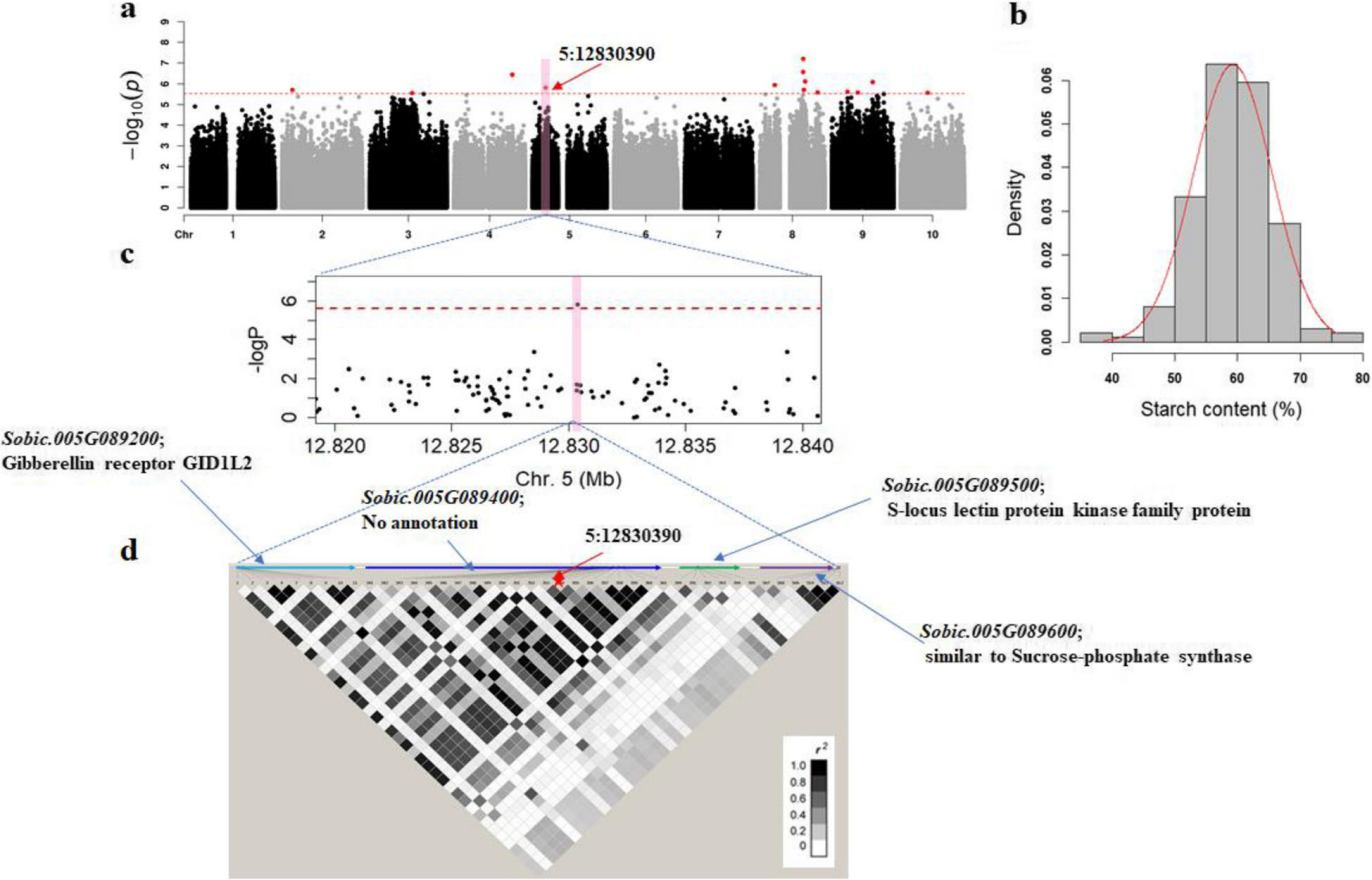
GWAS for starch content in sorghum grains (**a**) Manhattan plot for starch content GWAS. The red arrow shows significant SNP located close to candidate genes. (**b**) Distribution of starch content in 196 diverse accessions. (**c**) A close up of the significant association on chromosome 5. The broken red line represents the significance threshold. (**d**) LD block showing pairwise r^2^ values among all polymorphic sites in a candidate genes region, where the intensity of the colour corresponds to the r^2^ value as indicated on the legend

Most of the grain quality traits exhibited an approximately normal distribution of the frequency of phenotypic values as indicated by the skew values (Table 1) and histograms (for starch, see Fig. 4; for tannins see Fig. 3, and for amino acids see the diagonal of Fig. 2). The distribution of grain quality traits across the six sub-populations in our association panel was further investigated (Additional file 7: Table S5), which could provide fundamental knowledge for further germplasm utilization and improvement. The tannin content was highest in Pop4 (1.62%) and lowest in Pop1 and Pop5 (1.3%). Conspicuously, in Pop4, 83% (34/41) of the accessions were collected from China, where red sorghum grains are preferred for the production of Chinese *Baijiu* which derives a unique aroma from tannins [31]. Starch content showed no significant difference in accessions across the six sub-populations. Twelve amino acids showed significant differences in at least two populations whilst seven of them had no significant difference across populations.

Next, we investigated the phenotypic diversity of our accessions based on their usage (Additional file 1: Figure S1). The average tannin content was highest in the broom sorghum while starch content was highest in grain sorghum. Forage sorghum had the lowest level of starch in the grains. Majority of the amino acids had the highest levels in broom sorghum, while the highest levels of Met, Cys, Gly and Thr were found in grain and sweet sorghum.

### Association mapping and candidate genes identification

To dissect the genetic basis underlying the natural variation of grain quality traits in sorghum, we tested the association of each trait in 196 diverse accessions using 3,512,517 re-sequencing genome-wide SNPs (MAF > 0.05) based on FarmCPU model in MVP package of R [32]. The quantile-quantile plots showed that the principal components and relative kinships controlled the population structure effectively and reduced false positives to some extent, with no significant influence from the confounders. Given the overall linkage disequilibrium (LD) decay across the genome of this sorghum population at 40 kb (r^2^ = 2) (Fig. 1e), the significant SNPs within an 80-kb region flanking the left and right side of each significant SNP were considered to represent a locus. Candidate genes responsible for the variation of grain quality traits were scanned in the v3.1 of the *Sorghum bicolor* genome in Phytozome v.10 [33] based on this definition of a locus and listed in Additional file 8: Table S6.

### Tannin content

Genome-wide association analysis of tannin content in sorghum grains detected 14 SNPs representing 14 loci, and all of them were above the significance threshold (*P* ≤ 2.93E-06) (Fig. 3). The SNP with the strongest association with tannin content was 5:34971014 (*P* = 6.02E-12) which tagged *Sobic.005G110600* (32.4 kb away; similar to Glycosyl hydrolases family 18 protein). Also, one associated SNP 4:62341358 which was in high LD with previously cloned *Tannin1* gene in sorghum was included [21], although it was slightly below the significance threshold (*P* = 5.23E-6) (Fig. 3b). In the region of *Tannin1* gene, seven more candidate genes were identified (Fig. 3d and f; Additional file 8: Table S6). One of these 7 genes was a priori gene, *Sobic.004G281000,* (similar to MADS-box protein; ~ 10.1 kb from the significant SNP 4:62341358). It is a homologue to *TRANSPARENT TESTA 16* (*TT16)*, which plays a key role in tannins biosynthesis [34]. Two SNPs hit directly into candidate genes, namely, 4:61736881 (*P* = 1.62E-08), which is in the intron of *Sobic.004G273600* (RNA recognition motif) and a synonymous mutation 8:57291105 (*P* = 2.55E-08), in the exon of *Sobic.008G141833* (no annotation). Interestingly, 4:61736881 colocalized with a priori candidate gene *Sobic.004G273800* (~ 28.9 kb from the significant SNP), a Myb-related protein *Zm1* (Fig. 3d and e). This is consistent with previous result [26], albeit with a higher resolution. In future, evaluation of tannin content in multiple years and locations coupled with an increase in the sample size would further increase this resolution.

In addition, on chromosome 3 at ~ 57.7 Mb, SNP 3:57708223 (*P* = 1.08E-10) was in the region of the *R* locus, which controls the base pericarp colour (red, yellow or white) together with the *Y* locus [26]. However, the nearest gene *Sobic.003G230900*, and a putative homologue of *TRANSPARENT TESTA 3* (*TT3*; 68.8% protein similarity) [35], was ~ 667.6 kb from the significant SNP, outside our defined locus region.

Based on the KEGG online sorghum pathway database, at least six candidate genes were mapped into various metabolism pathways (Table 2). One of the candidate genes (*Sobic.009G072000*; ATP-dependent 6-phosphofructokinase 6) was involved in six metabolism pathways including pentose phosphate pathway, glycolysis/gluconeogenesis, RNA degradation, biosynthesis of amino acids, fructose and mannose metabolism, and galactose metabolism. And another intriguing candidate genes was *Sobic.004G273900*, encoding peroxidase 5, which was mapped on to the phenylpropanoid biosynthesis pathway and is the starting point for the production of flavonoids, including condensed tannins [37].

**Table 2:** Candidate genes for tannins and starch content that mapped into various KEGG pathways

Trait	SNP	Chr	Position (bp)^a^	*P*-value	candidate gene	Distance (kb)^b^	Annotation	Pathway^c^
Tannins	4:3635914	4	3,635,914	2.45E-06	Sobic.004G044200	1.01	1,4-dihydroxy-2-naphthoyl-CoA synthase, peroxisomal	Ubiquinone and other terpenoid-quinone biosynthesis
	4:61736881	4	61,736,881	1.62E-08	Sobic.004G273900	33.72	peroxidase 5	Phenylpropanoid biosynthesis
	5:34971014	5	34,971,014	6.02E-12	Sobic.005G110600	32.00	chitinase-3-like protein 1	Amino sugar and nucleotide sugar metabolism
	8:57291105	8	57,291,105	2.55E-08	Sobic.008G141700	2.38	heparanase-like protein 1	Glycosaminoglycan degradation
	9:8660880	9	8,660,880	1.22E-08	Sobic.009G072000	−26.21	phosphoribosylformylglycinamidine cyclo-ligase, chloroplastic/mitochondrial	Purine metabolism
	9:8660880	9	8,660,880	1.22E-08	Sobic.009G071800	−36.11	ATP-dependent 6-phosphofructokinase 6	Pentose phosphate pathway
	9:8660880	9	8,660,880	1.22E-08	Sobic.009G071800	−36.11	ATP-dependent 6-phosphofructokinase 6	Glycolysis/gluconeogenesis
	9:8660880	9	8,660,880	1.22E-08	Sobic.009G071800	−36.11	ATP-dependent 6-phosphofructokinase 6	RNA degradation
	9:8660880	9	8,660,880	1.22E-08	Sobic.009G071800	−36.11	ATP-dependent 6-phosphofructokinase 6	Biosynthesis of amino acids
	9:8660880	9	8,660,880	1.22E-08	Sobic.009G071800	−36.11	ATP-dependent 6-phosphofructokinase 6	Fructose and mannose metabolism
	9:8660880	9	8,660,880	1.22E-08	Sobic.009G071800	−36.11	ATP-dependent 6-phosphofructokinase 6	Carbon metabolism
	9:8660880	9	8,660,880	1.22E-08	Sobic.009G071800	−36.11	ATP-dependent 6-phosphofructokinase 6	Galactose metabolism
Starch	4:56136753	4	56,136,753	3.66E-07	Sobic.004G211866	15.24	S-adenosylmethionine decarboxylase proenzyme	Cysteine and methionine metabolism
	4:56136753	4	56,136,753	3.66E-07	Sobic.004G211866	15.24	S-adenosylmethionine decarboxylase proenzyme	Arginine and proline metabolism
	4:56136753	4	56,136,753	3.66E-07	Sobic.004G211833	8.31	cytochrome c oxidase subunit 6b-2	Oxidative phosphorylation

^a^ Physical position in base pairs for the peak SNP according to v3.1 of the sorghum genome

^b^ Distance of the gene from the significant SNP

^c^ Pathway of the candidate gene according to Kyoto Encyclopedia of Genes and Genomes (KEGG) database [36]

### Starch content

Using the starch content in sorghum grains of our diversity panel, 15 significant associations representing 14 loci were identified (Fig. 4). Significant loci were distributed across chromosomes 2, 3, 4, 5, 8, 9 and 10, and 4: 56136753 was the most significant SNP (*P* = 3.66E-07).

According to the definition of a locus (40 kb right and left of the significant SNP), 28 candidate genes in the LD decay distance of 5 loci represented by 5 SNPs were identified (Additional file 8: Table S6). Among the 5 SNPs, three hit directly on candidate genes. No candidate genes could be found within the locus region of 10 associated SNPs due to low density of genes in their regions. However, with the development of sequencing technologies, it is possible to identify candidate genes around these SNPs. Based on the compiled list of a priori candidate genes for starch content [7], at least one candidate gene encoding sucrose phosphate synthase (*Sobic.005G089600*) was identified ~ 22.8 kb away from associated SNP 5:12830390 (*P* = 1.53E-06) (Fig. 4). Furthermore, two candidate genes tagged by one SNP (4:56136753) were mapped into three KEGG metabolism pathways. These two genes included *Sobic.004G211866* that encodes S-adenosylmethionine decarboxylase proenzyme (involved in cysteine and methionine metabolism and arginine and proline metabolism) and *Sobic.004G211833* that encodes cytochrome C oxidase subunit 6B (involved in Oxidative phosphorylation).

### Amino acid content

**Fig. 5:**
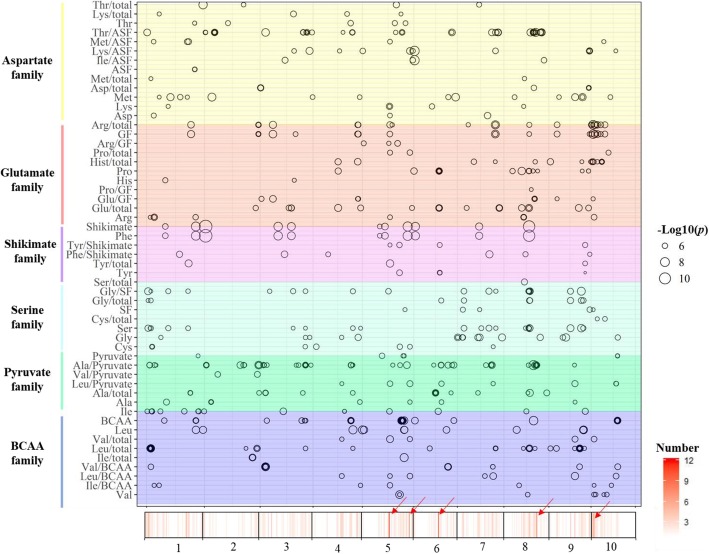
Chromosomal distribution of significant SNPs identified in amino acids content GWAS. SNP positions are represented by black circles. The size of the circle proportional to the significance level. Different amino acid families are represented by each colour as shown on the left of the *y*-axis. The *x-A*xis represents the physical position across the 10 sorghum chromosomes. The density map on the *x*-xis represents the number of amino acids significant loci identified across the genome. The red arrows show the association hotspots

In the GWAS of 17 amino acids and 44 derived traits, 711 SNPs representing 492 loci were identified (Fig. 5, Additional file 8: Table S6). Significant associations ranged from 0 in Glu to 60 SNPs in Leu/Pyruvate family. Furthermore, 47 SNPs representing 40 loci were detected in at least two amino acid traits, possibly due to tight gene linkages or pleiotropy of genes/loci (Fig. 5, Additional file 2: Figure S2). This was supported by strong correlations between several amino acid traits (Fig. 2) and may implicate candidate genes involved in the regulation of multiple amino acid traits. The full list of significant SNPs and potential candidate genes are presented in Additional file 8: Table S6, which could be used for further validation and investigation.

Through the curation of a priori candidate gene involved in amino acids biosynthesis and degradation from the gramene database, 698 genes were identified (Additional file 6: Table S4). Out of 698 a priori candidate genes, 34 were identified through GWAS signals (Table 3), which were distributed across 10 pathways/superpathways. BCAA family (Leu, Val, and Ile) and Aspartate family biosynthesis superpathways were overrepresented (17/34 genes). Interestingly, five loci that were identified in multiple amino acid traits hit directly into a priori candidate genes. For example, SNP 5:67881473, significantly associated with Ile/BCAA family, Val/BCAA family, Val/Pyruvate family and Val/Total, tagged *Sobic.005G194900* (similar to Phosphoserine phosphatase gene), a gene involved in BCAA family biosynthesis pathway. In addition, four direct hits of a priori candidate genes by GWAS signals were involved in more than one amino acid metabolism pathway. For example, at ~ 55.5 Mb on chromosome 10, SNP 10:55465480 significantly associated with Val/BCAA family tagged *Sobic.010G212000* (similar to Putative uncharacterized protein), a candidate gene involved in four pathways: arginine degradation I (arginase pathway), proline degradation I, proline degradation II and valine degradation I, which shows the pleiotropic nature of these candidate genes.

**Table 3:** Candidate genes for amino acid traits as identified by a priori candidate genes from amino acid biosynthesis and degradation pathways

Trait	SNP	Chr	Position (bp)^a^	candidate gene	Distance (kb)^b^	Annotation	Pathway^c^
Asp family	1:10068032	1	10,068,032	Sobic.001G127700	−25.64	similar to Lysine Decarboxylase, putative	lysine degradation I
Leu/BCAA	1:1014946	1	1,014,946	Sobic.001G011700	−4.06	similar to Aspartokinase	superpathway of lysine, threonine and methionine biosynthesis II
Val/BCAA	1:24852243	1	24,852,243	Sobic.001G241200	−21.77	similar to EDR1	threonine degradation III (to methylglyoxal)
Ile/BCAA	1:69010559	1	69,010,559	Sobic.001G405500	4.08	similar to Pyruvate decarboxylase isozyme 2	superpathway of leucine, valine, and isoleucine biosynthesis
Phe/Shikimate family	1:69010559	1	69,010,559	Sobic.001G405500	4.08	similar to Pyruvate decarboxylase isozyme 2	superpathway of leucine, valine, and isoleucine biosynthesis
Tyr/Shikimate	1:69010559	1	69,010,559	Sobic.001G405500	4.08	similar to Pyruvate decarboxylase isozyme 2	superpathway of leucine, valine, and isoleucine biosynthesis
Leu/BCAA	1:72963758	1	72,963,758	Sobic.001G453100	−10.87	similar to Homocysteine S-methyltransferase 1	superpathway of lysine, threonine and methionine biosynthesis II
Lys	2:13818293	2	13,818,293	Sobic.002G113600	15.98	similar to Rac GTPase activating protein 3-like protein	superpathway of lysine, threonine and methionine biosynthesis II
Ile/Asp family	2:4671226	2	4,671,226	Sobic.002G049200	−15.65	weakly similar to PHD finger transcription factor-like	superpathway of leucine, valine, and isoleucine biosynthesis
Thr/Asp family	2:58060555	2	58,060,555	Sobic.002G193800	15.95	GLUCOSE TRANSPORTER TYPE 1	superpathway of lysine, threonine and methionine biosynthesis II
Leu/Pyruvate	3:11583493	3	11,583,493	Sobic.003G126500	17.82	similar to Os01g0269000 protein	leucine degradation I
Ala/Pyruvate	3:3063590	3	3,063,590	Sobic.003G033900	26.43	similar to 1-aminocyclopropane-1-carboxylic acid synthase	phenylalanine degradation III
Ala/total	3:5411028	3	5,411,028	Sobic.003G061300	−17.63	Thiamine pyrophosphate dependent pyruvate decarboxylase family protein	superpathway of leucine, valine, and isoleucine biosynthesis
Leu/Pyruvate	3:57321213	3	57,321,213	Sobic.003G234701	12.80	similar to Pectin-glucuronyltransferase-like	arginine degradation I (arginase pathway)
Gly	3:70271670	3	70,271,670	Sobic.003G391600	9.40	similar to Putative 4-coumarate:coenzyme A ligase	superpathway of lysine, threonine and methionine biosynthesis II
Lys	4:11594929	4	11,594,929	Sobic.004G114500	−18.26	Core-2/I-branching beta-1,6-N-acetylglucosaminyltransferase family protein	glycine cleavage complex
Ser	4:1351183	4	1,351,183	Sobic.004G016800	−22.65	similar to Putative serine/threonine protein kinase	threonine degradation III (to methylglyoxal)
Thr/total	4:49321838	4	49,321,838	Sobic.004G156000	10.33	similar to Putative steroleosin	lysine degradation II
Leu/Pyruvate family	4:65472831	4	65,472,831	Sobic.004G319400	−16.93	similar to DNA helicase RECQE-like	superpathway of leucine, valine, and isoleucine biosynthesis
Val/BCAA	4:65472831	4	65,472,831	Sobic.004G319400	−16.93	similar to DNA helicase RECQE-like	superpathway of leucine, valine, and isoleucine biosynthesis
Glu/Glutamate family	5:3605534	5	3,605,534	Sobic.005G039700	10.91	similar to Rac GTPase activating protein 1	superpathway of lysine, threonine and methionine biosynthesis II
Pro	5:3605534	5	3,605,534	Sobic.005G039700	10.91	similar to Rac GTPase activating protein 1	superpathway of lysine, threonine and methionine biosynthesis II
Pro/Glutamate family	5:3605534	5	3,605,534	Sobic.005G039700	10.91	similar to Rac GTPase activating protein 1	superpathway of lysine, threonine and methionine biosynthesis II
Lys	5:5579891	5	5,579,891	Sobic.005G055300	–	similar to Tropinone reductase	lysine degradation II
Lys	5:5579891	5	5,579,891	Sobic.005G055300	–	similar to Tropinone reductase	phenylalanine degradation III
Lys	5:5579891	5	5,579,891	Sobic.005G055400	1.07	similar to Amidase family protein	arginine degradation X (arginine monooxygenase pathway)
Val/BCAA	5:63968450	5	63,968,450	Sobic.005G164200	2.49	similar to Putative uncharacterized protein	superpathway of leucine, valine, and isoleucine biosynthesis
Val/BCAA	5:63968450	5	63,968,450	Sobic.005G164300	6.84	similar to Putative uncharacterized protein	superpathway of leucine, valine, and isoleucine biosynthesis
Ile/BCAA	5:67881473	5	67,881,473	Sobic.005G194900	−22.93	similar to Phosphoserine phosphatase	superpathway of serine and glycine biosynthesis I
Val/Pyruvate	5:67881473	5	67,881,473	Sobic.005G194900	−22.93	similar to Phosphoserine phosphatase	superpathway of serine and glycine biosynthesis I
Val/BCAA	5:67881473	5	67,881,473	Sobic.005G194900	−22.93	similar to Phosphoserine phosphatase	superpathway of serine and glycine biosynthesis I
Val/total	5:67881473	5	67,881,473	Sobic.005G194900	−22.93	similar to Phosphoserine phosphatase	superpathway of serine and glycine biosynthesis I
Met/Asp family	5:69690963	5	69,690,963	Sobic.005G210500	20.74	similar to ATP-dependent DNA helicase, RecQ family protein, expressed	superpathway of leucine, valine, and isoleucine biosynthesis
Leu/BCAA	6:54237869	6	54,237,869	Sobic.006G187900	−0.29	similar to Acc synthase	phenylalanine degradation III
Leu/BCAA	6:54237869	6	54,237,869	Sobic.006G187900	−0.29	similar to Acc synthase	tyrosine degradation I
Tyr/total	7:60330803	7	60,330,803	Sobic.007G168200	−14.06	similar to Peptidyl-prolyl cis-trans isomerase	phenylalanine degradation III
Tyr/total	7:60330803	7	60,330,803	Sobic.007G168200	−14.06	similar to Peptidyl-prolyl cis-trans isomerase	tyrosine degradation I
Leu/Pyruvate	8:1074094	8	1,074,094	Sobic.008G012400	−27.01	similar to Os11g0142500 protein	superpathway of leucine, valine, and isoleucine biosynthesis
Ala/total	8:51569085	8	51,569,085	Sobic.008G111100	1.99	Predicted transporter (major facilitator superfamily)	superpathway of lysine, threonine and methionine biosynthesis II
Leu/Pyruvate	8:52368227	8	52,368,227	Sobic.008G114900	18.62	similar to Rac GTPase activating protein 3, putative, expressed	superpathway of lysine, threonine and methionine biosynthesis II
Leu/Pyruvate	8:59438201	8	59,438,201	Sobic.008G160700	−28.52	similar to Methylcrotonoyl-CoA carboxylase subunit alpha, mitochondrial precursor	leucine degradation I
Glu/Glutamate family	8:5993722	8	5,993,722	Sobic.008G057500	1.10	similar to Aldehyde dehydrogenase family protein	arginine degradation I (arginase pathway)
Pro/Glu family	8:5993722	8	5,993,722	Sobic.008G057500	1.10	similar to Aldehyde dehydrogenase family protein	arginine degradation I (arginase pathway)
Hist/total	10:6862967	10	6,862,967	Sobic.010G080300	−16.74	similar to Putative aminoacylase	superpathway of lysine, threonine and methionine biosynthesis II
Cys	10:8489698	10	8,489,698	Sobic.010G094900	9.71	similar to Putative uncharacterized protein	Tryptophan degradation III (eukaryotic)
Cys/total	10:8489698	10	8,489,698	Sobic.010G094900	9.71	similar to Putative uncharacterized protein	Tryptophan degradation III (eukaryotic)
Val/BCAA	10:55465480	10	55,465,480	Sobic.010G212000	25.56	similar to Putative uncharacterized protein	arginine degradation I (arginase pathway)
Val/BCAA	10:55465480	10	55,465,480	Sobic.010G212000	25.56	similar to Putative uncharacterized protein	proline degradation I
Val/BCAA	10:55465480	10	55,465,480	Sobic.010G212000	25.56	similar to Putative uncharacterized protein	proline degradation II
Val/BCAA	10:55465480	10	55,465,480	Sobic.010G212000	25.56	similar to Putative uncharacterized protein	valine degradation I

^a^ Physical position in base pairs for the peak SNP according to v3.1 of the sorghum genome

^b^ Distance of the gene from the significant SNP

^c^ Biosynthesis or degradation pathway of the candidate gene as curated from the Gramene pathway tool [38]

**Fig. 6:**
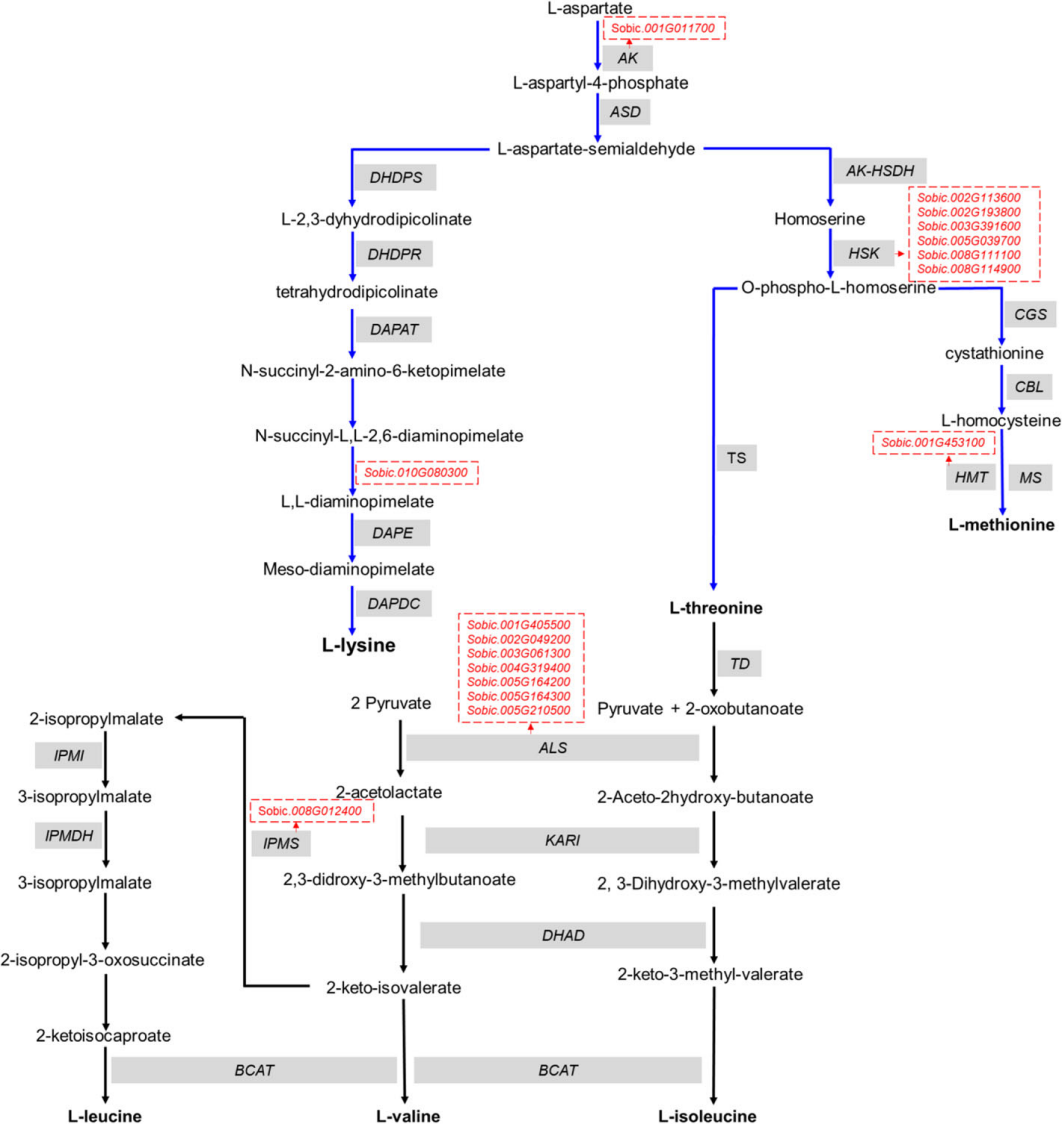
Biosynthesis of aspartate family and branched-chain amino acids. The blue and black arrows represent the aspartate family and branched-chain amino acid pathways, respectively. The candidate genes identified in this GWAS are shown in red text and surrounded by a textbox with broken red lines. AK, Aspartokinase; AK-HSDH, Aspartate kinase-homoserine dehydrogenase; ALS, Acetolactate synthase; ASD, Aspartate semialdehyde dehydrogenase; BCAT, branched-chain aminotransferases; CBL, cystathionine β-lyase; CGS, cystathionine γ-synthase; DAPAT, diaminopimelate aminotransferase; DAPDC, diaminopimelate decarboxylase; DAPE, diaminopimelate epimerase; DHAD, dihydroxylacid dehydratase; DHDPR, dihydrodipicolinate reductase; HMT, homocysteine S-methyltransferase; HSK, homo-Ser kinase; IPMDH, isopropylmalate dehydrogenase; IPMI, isopropylmalate isomerase; KARI, ketol-acid reductoisomerase; MS, Methionine synthase; TD, Threonine deaminase; TS, Threonine synthase

In conclusion, we integrated our GWAS results for a priori candidate genes identified for aspartate (8 candidate genes) and BCAA (9 candidate genes) family biosynthesis pathways based on published results in *Arabidopsis* [39, 40] (Fig. 6). *Sobic.001G011700* encodes Aspartokinase, an enzyme that catalyzes the conversion of Asp to β-aspartyl phosphate in the first step of the biosynthesis of Met, Lys and Thr, was identified. Six putative candidate genes (Table 3) were involved in the phosphorylation of homoserine kinase that converts homoserine to O-phospho-L-homoserine, a precursor for Met and Thr biosynthesis [39]. *Sobic.001G453100* encodes Homocysteine S-methyltransferase 1, an enzyme in the last step of methionine biosynthesis pathway and catalyzes transfer of methyl from S-methyl-L-methionine to L-homocysteine to yield H^+^ and 2 L-methionine.

Acetolactate synthase (ALS) catalyzes the first step of BCAA family biosynthesis pathway. ALS is involved in the conversion of two pyruvate molecules to 2-Acetolactate in the Val and Leu biosynthesis pathways or one pyruvate molecule and one 2-oxobutanoate molecule into 2-aceto-2-hydroxybutyrate in Ile biosynthesis pathway [40]. Seven of our GWAS candidate genes were homologues of ALS. Furthermore, 2-keto-isovalerate can be converted into 2-isopropylmalate with the help of Isopropylmalate synthase (IPMS) in the Leu biosynthesis pathway. We identified *Sobic.008G012400* (Tagged by SNP 8:1074094; *P* = 1.79E-06) in association with Leu/Pyruvate family (Table 3) that encodes 2-isopropylmalate synthase 1.

## Discussion

The success of a GWAS depends on the genetic variation in assembled association panel. The higher the diversity of the association panel, the higher the resolution of an association study in mining novel alleles [25]. Structure analysis of our association panel reflected classification of genotypes based on their geographical origin and type (i.e. landraces vs improved). Previous reports showed that the major genetic structure in sorghum was mainly according to racial and geographical origin classification [41]. However, botanical race information of the accessions in our association panel was limited. Furthermore, the PCA biplots showed similar clustering where PC1 to PC3 explained at least 34% of genetic variation, which was consistent with structure analysis for natural populations [41]. The decay rate in the average LD reflected the genetic variability among the accessions in different sub-populations of the association panel. Although the sub-populations with rapid LD decay rate (Pop2, Pop4 and Pop5) might be diverse based on type (landraces vs improved) and geographical origin, the small sample size in sub-populations with slow LD decay rate (Pop1, Pop3 and Pop6) may cause severe bias in LD decay estimation [42]. A decrease in LD to r^2^ = 0.2 at 40 kb for the whole population was within the range of previous studies which showed that the average LD decay rate in sorghum was between 15 kb and 150 kb [41, 43].

Sorghum is one of the major cereal crops with extensive genetic and phenotypic variations among existing germplasms. In this study, variations in tannins, starch and amino acids were investigated and most of these traits varied widely across our association panel, indicating the complexity of their respective biosynthetic processes. This variation in grain quality traits may be useful for further sorghum breeding. Our results showed that the levels of different amino acids were highly correlated, which may be due to the high interconnection of the metabolic clusters formed by amino acids, especially in the seed [11]. Furthermore, these correlations provided confidence in using extra traits derived from the absolute levels of amino acids. Previous GWAS on metabolites including amino acids showed that analyses of ratios derived from known biochemical interactions and correlation-based networks may result in stronger associations and more clear biological relevance compared to their absolute levels [11, 15]. In addition, human selection for different usage can influence the patterns of grain quality traits of different germplasms. For instance, our association panel, starch content was highest in grain and sweet sorghums. These materials are a potential source of genetic material for starch improvement in sorghum. Also, the environmental adaptations could lead to variations in grain quality traits, like in the case of tannins [41].

### Genetic control of tannins in sorghum

Flavonoid biosynthesis is mostly regulated at the transcriptional level [44]. The commonly identified transcriptional factors (TFs) that regulate flavonoid structural genes across plant species are those with MYB, basic helix-loop-helix (BHLH) domains and a WD40 protein (reviewed by [45]), known to work as an MYB-bHLH-WD40 (MBW) ternary transcriptional complex. However, by analyzing *Arabidopsis* mutants, more TFs with MADS-box [34], Zinc-finger [17], WRKY [46] domains, or homeodomain (HD) [47] and WD40 proteins [48], have been reported. Indeed, we identified potential candidates that encode TFs with these domains. For example, SNPs 2:2532818 tagged *Sobic.002G027401* and *Sobic.002G027300,* which encode a MADS-box protein and a C2H2-type zinc finger, respectively. On chromosome 4 at ~ 61.7 Mb, we identified a homologue of an MYB transcription factor *Zm1*_*,*_ which is homologous to C1 maize grain pigmentation gene [26]. *Tannin1* (*Sobic.004G280800*) gene that encodes a WD40 domain protein was identified at ~ 62.3 Mb on chromosome 4. *Sobic.004G281200,* colocalized with *Tannin1* gene and encodes a protein similar to scarecrow transcriptional regulator-like protein. Recently, SCARECROW-LIKE gene family TFs were reported to have an impact on several transcripts within the flavonoid pathway [49]. We propose further studies on the ~ 61.7 Mb and ~ 62.3 Mb QTL regions of chromosome 4 to elucidate potential genes and possible alternative ternary transcriptional complexes which contribute to tannin content variation in sorghum and plants species in general.

Using KEGG pathways, *Sobic.009G071800* that encodes ATP-dependent 6-phosphofructokinase 6 was mapped into multiple metabolism pathways, which include the pentose phosphate and glycolysis/gluconeogenesis pathways. The pentose phosphate and glycolytic pathways provide erythrose-4-phosphate and phosphoenolpyruvate, respectively, which are precursors to the shikimate pathway that provides phenylalanine. Phenylalanine is a precursor to phenylpropanoid metabolism that feeds various flavonoid pathways [50]. This putative candidate gene could be further studied to reveal its exact relevance in the flavonoid pathway.

### Candidate genes associated with grain starch as revealed by GWAS

In the current GWAS, 14 loci were associated with starch content. Identification of multiple loci shows the quantitative nature of starch content metabolism [39]. A peak at ~ 12.8 Mb of chromosome 5 tagged *Sobic.005G089600*, which encodes a sucrose phosphate synthase (SPS). SPS regulates the synthesis of sucrose and plays a significant role as a limiting factor in the export of sucrose out of the leaf [51]. SPS together with vacuolar acid invertases were shown to regulate sucrose fluxes in the sink tissues [52]. Also, high expression of *SPS1* in germinating seeds of rice suggested its role in conversion of starch or fatty acids into sucrose [53]. This candidate gene could be further used to study carbon partitioning which influences starch content in grains.

Based on the KEGG pathways, *Sobic.004G211866* (S-adenosylmethionine decarboxylase proenzyme) was mapped into four pathways of amino acids metabolism (cysteine, methionine, arginine and proline). S-adenosylmethionine decarboxylase is also known to be an essential enzyme of polyamine biosynthesis in plants, animals and microorganisms [54]. Polyamines include spermidine, spermine, and putrescine, which are considered as endogenous growth regulators involved in multiple processes of plant development such as grain filling and responses to biotic and abiotic stresses [55]. Polyamines were also found to mediate the effects of post-anthesis water deficiency on starch biosynthesis by regulating activities of soluble starch synthase (SS), granule-bound starch synthase (GBSS) and key enzymes in starch biosynthesis [56]. *Sobic.004G211866* is a proper candidate for genetic characterization to understand the importance of polyamines in determination of starch content in sorghum grains and their interaction with genes encoding mainstream starch biosynthesis enzymes (AGPase, SS, SBE, DBE, and GBSS).

### Candidate genes for amino acids in the sorghum association panel

Besides their importance as building blocks for proteins, amino acids as secondary metabolites also act as molecular signals during germination, growth, development and reproduction [12]. Genetic control of amino acids biosynthesis and degradation remains poorly understood in higher plants. We identified 492 loci associated with 17 amino acids and their derived traits (Additional file 8: Table S6). Numerous candidate genes identified did not directly associate with known amino acid traits. Although a number of them are likely to be false positive associations, several of them may be novel associations that are yet to be discovered as causal genes for amino acids variation, making our GWAS results an entry point for further studies. However, previously characterized genes were identified. For instance, two putative homologs of *opaque1* [57], *Sobic.001G257800* and *Sobic.002G339300* colocalized with significantly associated SNPs, 1:30450051 (Cys and Serine family) and 2:70633375 (Val/Total), respectively. *Opaque1* encodes a myosin XI protein which plays an important role in endoplasmic reticulum motility and protein body formation in the endosperm [57]. A homolog of *Opaque2 (O2)* gene [58], *Sobic.001G056700* was ~ 12 kb from SNP 1:4291408, significantly associated with Leu/Pyruvate (*P* = 1.07E-06). *O2* encodes a bZIP transcription factor whose mutant (*o2*) is characterized with almost two-fold increase in essential amino acids, especially Lys and Trp.

Using a compiled list of a priori candidate genes involved in amino acid biosynthesis and degradation, 8 candidate genes encode 3 enzymes in the aspartate pathway were identified. They included one aspartokinase gene, six homoserine kinase genes, and one homocysteine *S*-methyltransferase gene. Animals and humans cannot synthesize aspartate-derived amino acids, so they are called essential amino acids and must be obtained through dietary intake. However, cereals that make majority of the diet worldwide are deficient in aspartate-derived amino acids [15]. Manipulation of the aspartate-derived amino acids biosynthetic pathway may be an alternative approach for plant breeders and agricultural biotechnologists to increase essential amino acids content in cereals, including sorghum.

Branched-chain amino acids (BCAA) is comprised of three essential amino acids, including Val, Leu and Ile that are biochemically related, with branched hydrocarbon side chains responsible for their aliphatic nature [40]. BCAA can act as signaling molecules, and their supplementation in animals prevents oxidative damage and skeletal muscle mitochondrial biogenesis [10]. Our GWAS identified eight candidate genes associated with BCAA biosynthetic pathway, seven of which were involved in the acetolactate synthase (ALS) reaction. ALS is a target site for five herbicide chemical groups: sulfonylurea, imidazolinone, triazolopyrimidine, pyrimidinyl-thiobenzotes, and sulfonyl-aminocarbonyl-triazolinone. A significant SNP 3:5411028 was identified in the vicinity of one of ALS encoding homologs -*Sobic.003G061300* (~ 17.6 kb from the SNP), which encodes a thiamine pyrophosphate dependent pyruvate decarboxylase family protein. Binding of the herbicide was shown to induce progressive damage or modification to Thiamine diphosphate (ThDP), a cofactor for ALS activity [59]. Therefore, *Sobic.003G061300* could potentially be used for further studies on the role of amino acids in herbicide development. Perhaps the most intriguing candidate gene in BCAA biosynthetic pathway is *Sobic.008G012400* (encodes 2-isopropylmalate synthase), tagged by SNP 8:1074094 (*P* = 1.79E-06, ~ 27 kb downstream of significant SNP), associated with Leu/Pyruvate family. Isopropylmalate synthase (IPMS, EC 2.2.3.13) catalyzes condensation of 3-methyl-2-oxobutanoate and acetyl-CoA, resulting in 2-isopropylmalate [40]. ALS and IPMS work together to maintain homeostasis of Val and Leu [60]. While ALS affects the flux of Val and Leu into their pathways, IPMS regulates their partitioning. Candidate genes for ALS and IPMS could be further studied to manipulate BCAA metabolism.

Degradation of amino acids contributes to the maintenance of energy state of the cell during stress tolerance as well as regulates their levels in plants [39, 40]. For instance, BCAA catabolism supports respiration, acts as an energy source during oxidative phosphorylation and a detoxification pathway during plant stress, donates electrons to the electron transport chain in the mitochondria and synthesize aroma volatiles in fruits [10]. In our GWAS, homologues of two enzymes involved in Leu degradation: *Sobic.003G126500* (encoding Hydroxymethylglutaryl-CoA lyase) and *Sobic.008G160700* (encoding Methylcrotonoyl-CoA carboxylase subunit alpha, mitochondrial precursor) were identified. Hydroxymethylglutaryl-CoA lyase is a vital enzyme in the last step of leucine catabolism, ketogenesis, and mitochondrial Methylcrotonoyl-CoA carboxylase catalyzes the fourth step of Leu catabolism in mammals and higher plants [40]. In *Arabidopsis*, mutants of Hydroxymethylglutaryl-CoA lyase (*hml1–1*, and *hml1–2*) and Methylcrotonoyl-CoA carboxylase (*mcca1–1* and *mccb1–1*), showed elevated accumulation of Ile, Leu and Val in mature seeds despite the presumptive specific role of the two enzymes to Leu degradation [61]. Surprisingly, the mutants also accumulated biosynthetically unrelated amino acids such as His and Arg in the seeds, more than the wild type, hence a proof of complex interconnection of amino acid networks.

## Conclusion

Based on high-density re-sequencing data and robust statistical analysis, we were able to identify genetic regions previously associated with grain quality traits including homologs of *Tannin1*, *Zm1* and *TT16* for tannins content, sucrose phosphate synthase (*SPS*) for starch content and *opaque1* and *opaque2* for amino acids. We also identified novel candidate genes that mapped into various metabolic pathways associated with tannins, starch and amino acids. For amino acids, we reconstructed aspartate and BCAA biosynthesis pathways which contribute to six essential amino acids using 15 candidate genes identified in this GWAS. These identified candidate genes could be further verified and fine mapped using biparental populations. Furthermore, the putative candidate genes will be the genesis of genomics-assisted breeding for improvement of sorghum grain nutritional quality.

## Methods

### Plant materials

A total of 196 diverse sorghum accessions were collected for the evaluation of grain quality traits based on their stem characteristics (dry, pithy or juicy), type (landraces or improved), usage (sweet, grain, forage or broom sorghums), and geographical centres of collection and localities (Additional file 3: Table S1). All the 196 inbred lines were planted in the experimental field of Institute of Botany, Chinese Academy of Sciences (IBCAS) (Beijing; N40°, E116°, altitude 112.07 m) in late April of 2015. The standard agricultural practice was followed for optimum plants growth and development, including irrigation, fertilizer application and pest control. Mature grains of each inbred line were harvested and bulked for tannins, starch and amino acid levels analysis.

### Measurement of amino acids

The amino acid contents of mature sorghum grains from each of the 196 diverse inbred lines were determined by hydrolysis/high-performance liquid chromatography and ultraviolet spectrophotometry (HPLC-UV) method. 20 mg of grain flour of each sample was used for amino contents determination. 1 mL of 6 M HCl was added to each sample and hydrolyzed at 110 °C for 24 h. The suspension was centrifuged at 12000×*g* for 10 min and 100μLof the supernatant decanted and dried in vacuum. The dried hydrolysate was re-dissolved in 1 mL 0.1 M HCl and centrifuged at 12000×*g*. Subsequently, 1 μL liquid supernatant was separated by analytical column ZORBAX Eclipse-AAA (Agilent, 5 μm, 4.6 × 250 mm) and analyzed by HPLC-UV System (1260, Agilent Technologies, USA). The content of each of the 17 amino acids in every sample was expressed as nmol mg^− 1^ of dry grain flour. The amino acid data used for association analysis were the mean values of three biological replicates. The absolute levels of amino acids determined included those of Ala = Alanine, Arg = Arginine, Asp = Aspartate, Cys = Cysteine, Glu = Glutamate, Gly = Glycine, His = Histidine, Ile = Isoleucine, Leu = Leucine, Lys = Lysine, Met = Methionine, Phe = Phenylalanine, Pro = Proline, Ser = Serine, Thr = Threonine and Val = Valine. Relative levels of amino acids were calculated from the absolute levels as follows: (a) The sum of absolute levels of amino acids (Total), (b) The relative level of each amino acid as a percentage of the Total; e.g. Ile/Total, (c) The sum of amino acids in the same biochemical family (For instance, branched-chain amino acids (BCAA include, Ile, Leu and Val)), (d) Ratio of each absolute amino acid to sum of its biochemical family; e.g. Ile/BCAA.

### Tannins content determination

A modified International Standardization Organization [62] method was used to determine the tannin content in sorghum grains. Milled 200 mg of sorghum grain flour was dissolved in 10 mL 75% dimethylformamide (DMF) solution for 1 h at room temperature, with vortex mixing at 5 min interval. The solution was centrifuged, the supernatant removed and preserved in the dark. The supernatant was divided into two aliquots: test tube 1 and 2. In test tube 1, distilled water and ammonia solution were added and thoroughly mixed on a vortex before incubation at 25–30 °C for 10 min. The absorbance value A1 of the sample solution was measured with a spectrophotometer at a wavelength of 525 nm. In test tube 2, distilled water, ferric ammonium citrate solution and ammonia solution were added, thoroughly mixed, and then incubated at 25–30 °C for 10 min. The absorbance value A2 of the sample solution in test tube 2 was measured at 525 nm with water as a blank. The tannin content was determined using a calibration curve prepared using tannic acid on dry weight basis:

$$ \mathrm{Tannin}\ \mathrm{content}\ \left(\%\right)=\frac{0.671\left(\mathrm{A}2-\mathrm{A}1\right)+0.131}{\mathrm{W}} $$

In the formula, *W* was the dry weight of each sample (0.2 g), 0.131 was a conversion factor generated from the tannic acid standard curve.

### Determination of starch content in sorghum grains

Starch content of each of the 196 diverse accessions was estimated in triplicate through Amylogulosidase-α-amylase technique of Association of Official Agricultural Chemists [63] with some modifications. 30 mg of milled sorghum sample was weighed into centrifuge tubes, 0.7 mL 80% ethanol added and mixed, incubated in a water bath at 70 °C for 2 h with frequent mixing every 15 min, then centrifuged at 12000×g for 10 min. The supernatant was discarded and the precipitate mixed with 80% ethanol and thoroughly stirred on a vortex mixer. 1 mL of thermostable α-amylase was added and incubated in boiling water for 10 min, and glucosidase was subsequently added after cooling, then incubated at 50 °C for 30 min, centrifuged at 3000 g for 10 min and then the supernatant was collected into a new tube. Glucose oxidase-peroxidase-aminoantipyrine buffer mixture was added to the supernatant and incubated at 50 °C for 30 min. The optical density (OD) was measured on a spectrophotometer (Beckman Coulter) as absorbance at 510 nm. The starch content was expressed as starch % w/w (dry weight basis) and the starch content used for GWAS was the average value from three biological replicates.

### Genotype data

To identify nucleotide polymorphisms for diversity evaluation and GWAS, whole-genome re-sequencing of 196 accessions was performed. The re-sequencing and SNP detection pipeline were as described in our previous study [64]. In brief, sequencing was done on the Illumina Hiseq X Ten platform by pair-end sequencing at an average depth of approximately 5.67×. Adapters were trimmed, and low quality reads filtered before mapping the clean reads to BTx623 (v3.1) reference genome using Burrows-Wheeler Alignment software (BWA, v.0.7.8) [65]. SNPs were called independently using the Genome Analysis Toolkit (GATK, Ver. 3.1, HaplotypeCaller) [66] and SAMtools (Ver. 1.3) package [67]. A set of common variations detected by both tools were extracted with a strict filtration procedure then used as known sites following BQSR (recalibrating the base quality score) method embedded in GATK. HaplotypeCaller in GATK was used to detect variations, and then a VQSR (variant quality score recalibration) procedure was followed. In total, 40,315,415 SNP markers were identified across 196 diverse accessions.

Before performing GWAS, the SNPs were further filtered according to the following criteria: (a) deleted SNPs in the scaffolds, (b) removed SNPs with > 20% missing rate, (c) retained SNPs with at least 5% minor allele frequency (MAF).

### Population structure, phylogeny and linkage disequilibrium

Population structure was estimated using the ADMIXTURE program, a high-performance tool for estimation of ancestry in unrelated individuals using a maximum likelihood method [29]. A total of 841,038 SNPs (r^2^ < 0.2) distributed across the genome were identified after filtration with PLINK [68] to minimize LD and used in the analysis of population structure. To choose the actual number of ancestral populations, ADMIXTURE was run with a 10-fold cross-validation procedure for K = 2 to K = 10 and the K value with the lowest standard error was selected [29]. Further, to summarize the genome-wide variation in the association panel, principal component analysis (PCA) was performed in GCTA software [69]. The first two principal components were plotted in R software [70] based on the six subpopulations identified in ADMIXTURE, to visualize the population stratification.

The phylogenetic analysis was conducted based on the SNP data in SNPhylo (Ver. 20,140,701) [30]. In SNPhylo, an automated Bash shell script *snphylo.sh* was implemented with additional options: -p 5 -c 2 -l 0.2 -m 0.05 -M 0.5 -A -b -B 1000. Where, *p 5* is the percentage of low coverage samples (5%); *c 2* is the minimum depth of coverage [2], *l 0.2* is the linkage disequilibrium (LD) (0.2); *m 0.05* is the minor allele frequency (MAF) of 0.05; *M 0.5* is the maximum missing rate of 50%; *A* is for performing multiple alignments by MUSCLE; *−b –B 1000* is a command to perform non-parametric 1000 bootstrap analysis. The phylogenetic tree was visualized and annotated using the Interactive Tree of life [71].

The extent of LD decay in the association panel was calculated for all SNPs using Haploview [72], where pairwise comparisons among all SNP markers (MAF > 0.05) were calculated in an intra-chromosomal maximum distance of 500 kb to obtain the r^2^ values. The averages of r^2^ values for the whole population and all the six sub-populations, across each of the 10 sorghum chromosomes were plotted against the distance of the polymorphisms using an in-house R script. The smooth.spline function was integrated into the R-script to estimate the LD decay simulation curves.

### Association mapping and candidate gene selection

Genome-wide association analysis on tannins, starch content and amino acids in sorghum grains, was performed with FarmCPU model [32] implemented in the R-package MVP (A Memory-efficient, Visualization-enhanced, and Parallel-accelerated Tool for Genome-Wide Association Study)(https://zzlab.net/FarmCPU). The top three principal components were fitted as covariates to account for population structure. The kinship matrix was internally calculated within the MVP package using VanRaden method [73] and then combined with the population structure to control for Type I error. A Bonferroni-like multiple test correction, as described by [74], was used to determine the threshold for detecting significant associations. In brief, instead of 3,512,517 independent tests equivalent to the number of SNPs used for GWAS, the average extent of LD across the genome was used to estimate the total number of tests. Independent tests were estimated as: Total chromosomes’ length (683,645,045 bp) divided by the average LD decay distance of our association panel (40,000 bp) to get 17,091.13 tests. With 0.05 as the desired probability of type I error, a significance threshold was calculated as 0.05/17,091.13 = 2.93E-06.

Candidate genes were identified and annotated from v3.1 of the sorghum genome in Phytozome v.10 [33]. All the genes within an 80 kb window (40 kb upstream and 40 kb downstream of significant SNP), were identified as potential candidate genes based on the average LD decay distance of our diversity panel.

### Co-localization of GWAS candidate genes with genes related to grain quality traits

Sets of potential candidate genes that were previously characterized or associated with grain quality traits were compiled. For tannin and starch sets, we used the prior compiled lists by [26] and [7], respectively. In brief, based on the fact that most of the flavonoid genes are conserved across diverse plant species, orthologs of *Arabidopsis* were compiled as a priori genes for tannin content. Two cloned flavonoid genes in sorghum, *Yellow seed1* [75] and *Tannin1* [21], were also included. The list of a priori genes for starch content was compiled based on candidate genes involved in grain composition, grain maturation, and grain filling [7]. We curated a priori candidate genes involved in sorghum amino acids metabolism using the Gramene pathway tool [38] (Additional file 6: Table S4). During the curation process, genes in the pathways and superpathways of amino acids biosynthesis and degradation were included. Furthermore, for the identification of genes encoding starch and tannin metabolism-related enzymes, candidate genes were mapped into the Kyoto Encyclopedia of Genes and Genomes (KEGG) pathways database [36].

## Supplementary information

**Additional file 1: Figure S1.** A radar chart showing the distribution of average values of grain quality traits across different sorghum usage groups. The numbers on the chart are the average values of each grain quality trait, and the length of lines is proportional to these averages. Different line colours represent different usage groups.

**Additional file 2: Figure S2.** Significant loci detected in multiple amino acid traits. A total of 47 SNPs representing 40 loci were identified in at least two amino acid traits. All SNPs within a 40 kb region defines a locus.

**Additional file 3: Table S1.** List of 196 worldwide accessions used in this study.

**Additional file 4: Table S2.** The mean values of 17 amino acids, tannins and starch.

**Additional file 5: Table S3.** Lists of amino acids, absolute and derived traits calculated from the sum of all amino acids and their biochemical interactions.

**Additional file 6: Table S4.** 698 a priori candidate genes in the proteinogenic amino acids biosynthesis and degradation pathway.

**Additional file 7: Table S5.** Variation of grain quality traits across six subpopulations of the association panel.

**Additional file 8: Table S6.** The list of total candidate genes detected by grain quality traits’ GWAS.

## Data Availability

The data supporting our findings are presented in additional files.

## Abbreviations

AGPase: ADP-glucose pyrophosphorylases
ALS: Acetolactate synthase
BCAA: Branched-chain amino acids
BHLH: Basic-helix-loop-helix
BWA: Burrows-wheeler alignment software
DBE: Starch debranching enzyme
GATK: Genome analysis toolkit
GBSS: Granule-bound starch synthase
GWAS: Genome-wide association study
HMT: Homocysteine-S-methyltransferase
HSK: Homoserine kinase
IPMS: Isopropylmalate synthase
KEGG: Kyoto encyclopedia of genes and genomes
LD: Linkage disequilibrium
MAF: Minor allele frequency
PCA: Principal component analysis
QTL: Quantitative trait loci
SBE: Starch branching enzyme
SCL8: Scarecrow-like 8
SNP: single nucleotide polymorphism
SPS: Sucrose phosphate synthase
SS: Starch synthase
TFs: Transcriptional factors
ThDP: Thiamine diphosphate
