# Supplementary figures and images for: Genome-wide association study reveals that different pathways contribute to grain quality variation in sorghum (Sorghum bicolor)
Source: BMC Genomics. 2020 Jan 31;21:112. doi: 10.1186/s12864-020-6538-8 (PMC6995107; doi:10.1186/s12864-020-6538-8)

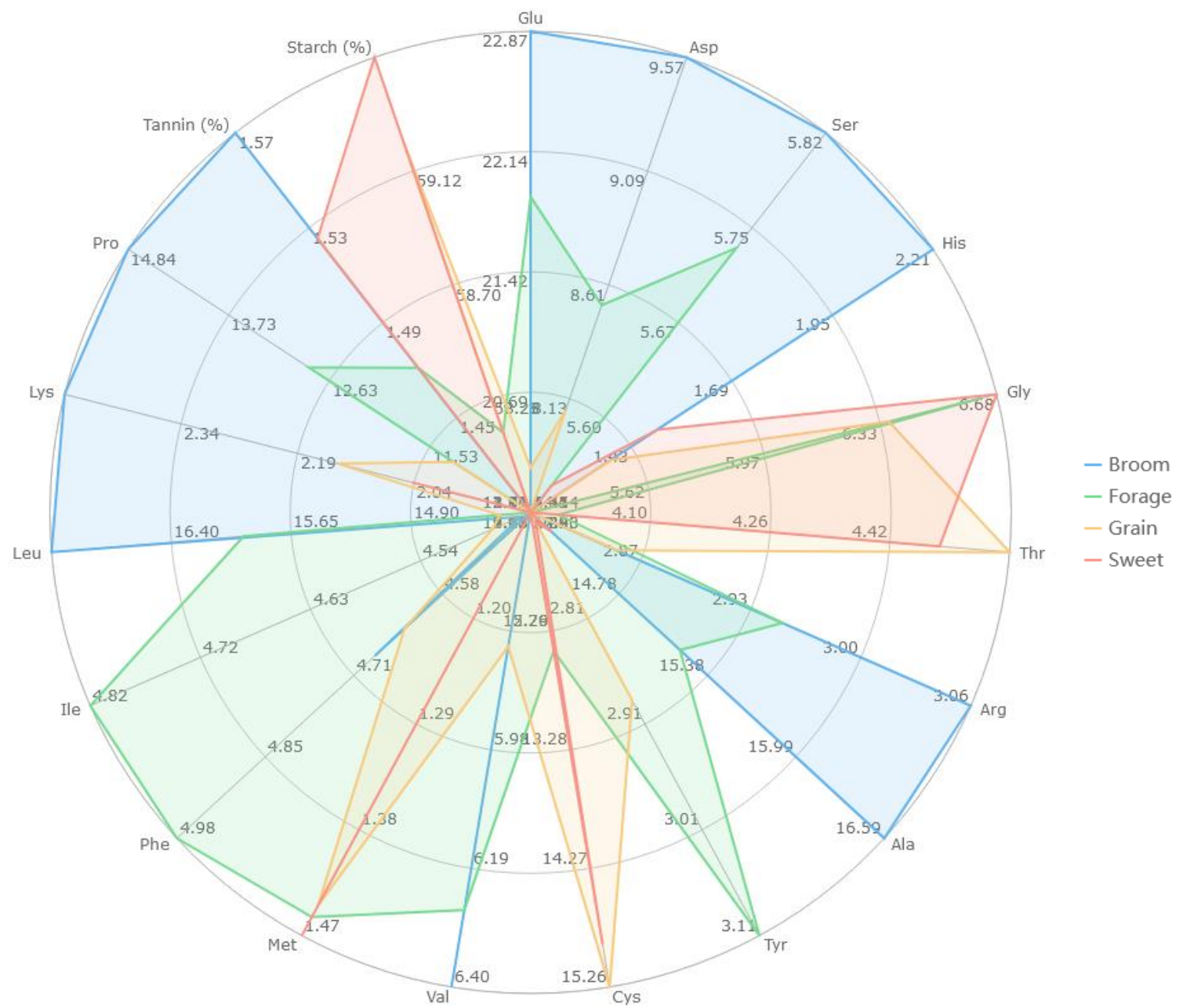

Supplement: Supplementary file 1 — Additional file 1: Figure S1. A radar chart showing the distribution of average values of grain quality traits across different sorghum usage groups. The numbers on the chart are the average values of each grain quality trait, and the length of lines is proportional to these averages. Different line colours represent different usage groups. [file 12864_2020_6538_MOESM1_ESM.pdf]
