# Supplementary material for: Genome-wide association study reveals that different pathways contribute to grain quality variation in sorghum (Sorghum bicolor)
Source: BMC Genomics. 2020 Jan 31;21:112. doi: 10.1186/s12864-020-6538-8 (PMC6995107; doi:10.1186/s12864-020-6538-8)
Supplement: Supplementary file 2 — Additional file 2: Figure S2. Significant loci detected in multiple amino acid traits. A total of 47 SNPs representing 40 loci were identified in at least two amino acid traits. All SNPs within a 40 kb region defines a locus. [file 12864_2020_6538_MOESM2_ESM.pdf]

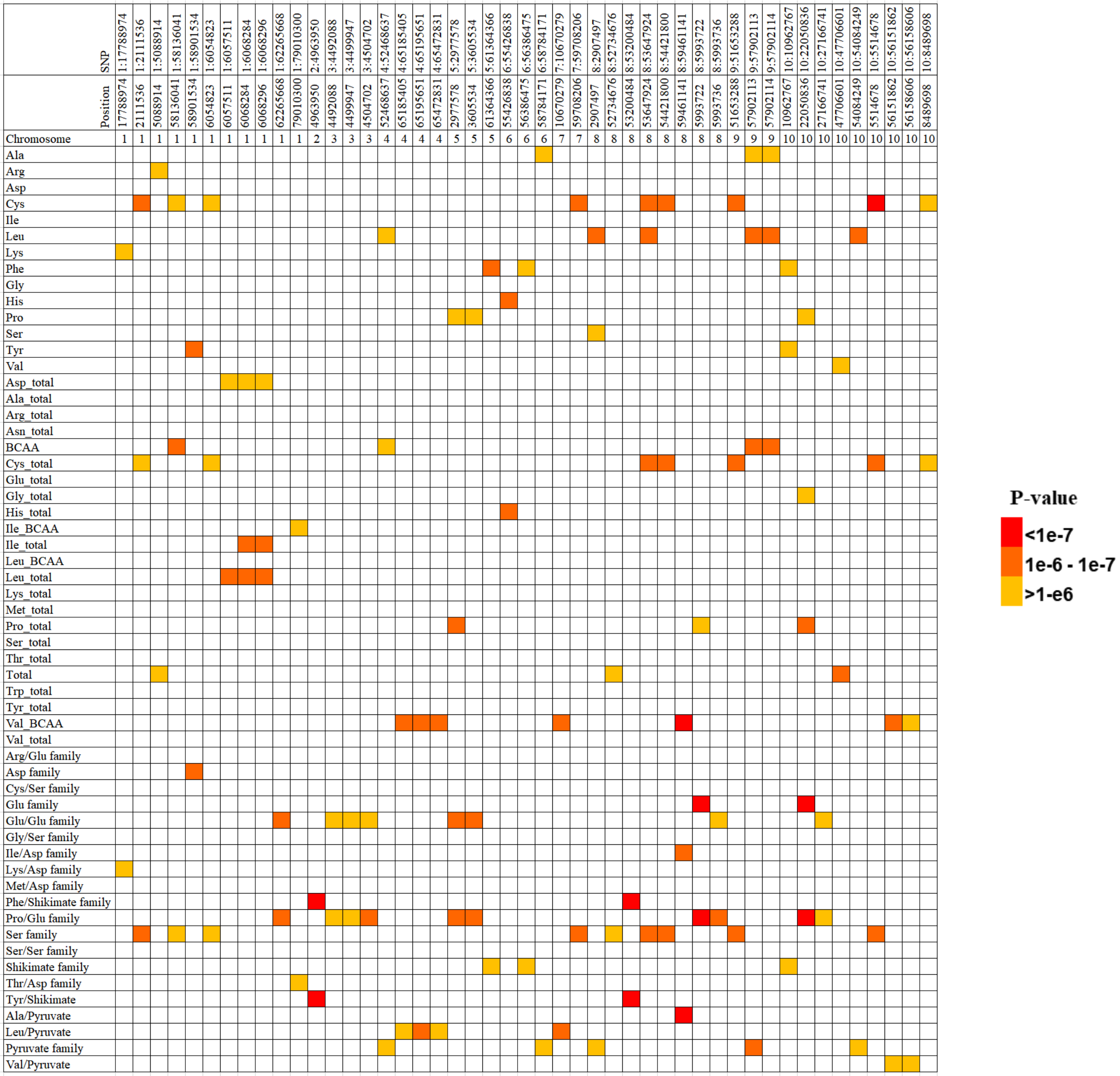

SNP
1:17788974
1:2111536
1:5088914
1:58136041
1:58901534
1:6054823
1:6057511
1:6068284
1:6068296
1:62265668
1:79010300
2:4963950
3:4492088
3:4499947
3:4504702
4:52468637
4:65185405
4:65195651
4:65472831
5:2977578
5:3605534
5:61364366
6:55426838
6:56386475
6:58784171
7:10670279
7:59708206
8:2907497
8:52734676
8:53200484
8:53647924
8:54421800
8:59461141
8:5993722
8:5993736
9:51653288
9:57902113
9:57902114
10:10962767
10:22050836
10:27166741
10:47706601
10:54084249
10:5514678
10:56151862
10:56158606
10:8489698
Position
17788974
2111536
5088914
58136041
58901534
6054823
6057511
6068284
6068296
62265668
79010300
4963950
4492088
4499947
4504702
52468637
65185405
65195651
65472831
2977578
3605534
61364366
55426838
56386475
58784171
10670279
59708206
2907497
52734676
53200484
53647924
54421800
59461141
5993722
5993736
51653288
57902113
57902114
10962767
22050836
27166741
47706601
54084249
5514678
56151862
56158606
8489698
Chromosome
1 1 1 1 1 1 1 1 1 1 1 2 3 3 3 4 4 4 4 5 5 5 6 6 6 7 7 8 8 8 8 8 8 8 8 9 9 9 10 10 10 10 10 10 10 10 10
Ala
Arg
Asp
Cys
Ile
Leu
Lys
Phe
Gly
His
Pro
Ser
Tyr
Val
Asp_total
Ala_total
Arg_total
Asn_total
BCAA
Cys_total
Glu_total
Gly_total
His_total
Ile_BCAA
Ile_total
Leu_BCAA
Leu_total
Lys_total
Met_total
Pro_total
Ser_total
Thr_total
Total
Trp_total
Tyr_total
Val_BCAA
Val_total
Arg/Glu family
Asp family
Cys/Ser family
Glu family
Glu/Glu family
Gly/Ser family
Ile/Asp family
Lys/Asp family
Met/Asp family
Phe/Shikimate family
Pro/Glu family
Ser family
Ser/Ser family
Shikimate family
Thr/Asp family
Tyr/Shikimate
Ala/Pyruvate
Leu/Pyruvate
Pyruvate family
Val/Pyruvate
P-value
<1e-7
1e-6 - 1e-7
>1-e6
